# Effective Pain Management of Postherpetic Neuralgia Using a Combination of Analgesics and Conservative Measures

**DOI:** 10.7759/cureus.73132

**Published:** 2024-11-06

**Authors:** Takayoshi Tsubaki, Erina Kodaka, Yuhei Kitano, Maiko Kodama, Shintaro Fukumoto, Chihaya Takano, Shiro Iino, Yasuo Hirono

**Affiliations:** 1 Surgery, Cancer Care Promotion Center, University of Fukui Hospital, Fukui, JPN; 2 Dermatology, Faculty of Medical Sciences, University of Fukui, Fukui, JPN; 3 Palliative Care, Cancer Care Promotion Center, University of Fukui Hospital, Fukui, JPN; 4 Neuropsychiatry, Faculty of Medical Sciences, University of Fukui, Fukui, JPN; 5 Nursing, University of Fukui Hospital, Fukui, JPN

**Keywords:** duloxetine hydrochloride, hot compress, opioids, oxycodone hydrochloride, postherpetic neuralgia, pregabalin

## Abstract

Postherpetic neuralgia (PHN) is characterized by persistent pain following the resolution of a herpes zoster rash. PHN is often resistant to treatment and can significantly reduce the patient’s quality of life. Effective symptom relief is crucial and various treatments, including pharmacotherapy, have been attempted. Given that symptoms can persist for a prolonged period, they can substantially affect the physical and mental well-being of the patients.

A 73-year-old man developed herpes zoster while undergoing treatment for a head angiosarcoma. Despite the resolution of the rash, the pain persisted, leading to the diagnosis of PHN. Treatment was initiated with a range of medications, including mecobalamin, pregabalin, and a combination of tramadol and acetaminophen, along with general pain relievers standardized as WHO Step 1 medications, which include acetaminophen and non-steroidal anti-inflammatory drugs (NSAIDs). However, achieving adequate pain control is challenging and results in frequent hospitalizations. Due to the patient’s depression and the concurrent use of a selective serotonin reuptake inhibitor, duloxetine hydrochloride could not be prescribed. Instead, opioid therapy with continuous fentanyl citrate infusion was initiated. Eventually, the treatment was switched to oxycodone hydrochloride, which successfully stabilized the patient’s symptoms. The use of conservative measures such as hot compresses also contributes to symptom relief. Alleviating pain symptoms using a combination of pharmacotherapeutic and non-pharmacological treatments is extremely important.

## Introduction

Herpes zoster is caused by the reactivation of latent varicella-zoster virus (VZV) in the cranial and dorsal root ganglia owing to a decrease in specific cellular immunity against VZV. The rash typically appears two to three days later along a single unilateral skin segment [1]. Although the rash generally improves within a few weeks, pain may persist after healing, leading to postherpetic neuralgia (PHN), a long-lasting neuropathic pain syndrome arising from peripheral nerves affected by the herpes zoster virus. Postherpetic neuralgia may be resistant to various therapies, including pharmacotherapy, and has a significant psychological and physical impact on patients, affecting their quality of life (QOL) [1-3].

There has been no established treatment [2], and multiple approaches have been attempted to alleviate the distressing symptoms in patients with PHN [2,3]. The medications included tricyclic antidepressants, antiepileptic drugs, and opioids. Psychosocial support and invasive procedures, such as nerve blocks and intrathecal injections, have also been tested [3]. Because pain lasts for at least 90 days, optimizing pain relief is necessary to prevent reduction in the patient’s quality of life [2].

The patient in this case had underlying depression, was unable to express the distress symptoms he was experiencing, and was limited to the medications he could use. He was also a patient in the terminal stage of cancer who wanted to avoid invasive procedures.

The patient was in the terminal stages of cancer and was difficult to treat invasively. Additionally, pre-existing depression limited medication modification. The patient also developed postherpetic neuralgia during treatment for head angiosarcoma, which was effectively managed with a combination of pharmacological and non-pharmacological therapies.

## Case presentation

**Figure 1:**
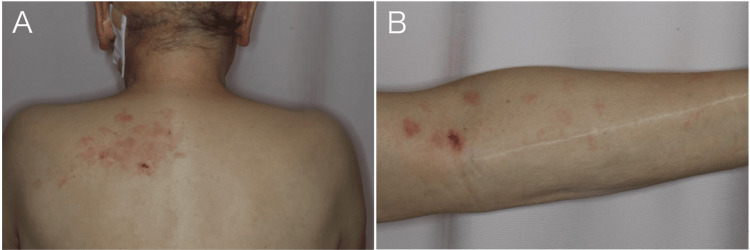
Physical examination on admission. (A) Examination of the patient’s back revealed a cluster of vesicles and erythema, predominantly on the upper left side. (B) The left upper limb showed scattered erythema, predominantly on the anterior elbow.

A 73-year-old man was referred to our hospital for chemotherapy for angiosarcoma of the head with multiple lung, liver, and cervical lymph node metastases. At presentation, a cluster of vesicles and erythema were observed extending from the left upper back (Figure 1A) to the left upper limb (Figure 1B).

The patient was diagnosed with a herpes zoster infection. There was no history of chicken pox. We initially commenced treatment with amenamevir (400 mg/day) and mecobalamin (1500 μg/day); the rash crusted over in approximately one week. However, the pain persisted and the patient was diagnosed with postherpetic neuralgia. Pain-relieving treatment was initiated.

Our patient had a history of depression and was regularly taking escitalopram oxalate (a selective serotonin reuptake inhibitor {SSRI}, 20 mg/day), aripiprazole (an antipsychotic, 1.5 mg/day), trazodone hydrochloride (an antidepressant, 25 mg/day), and lemborexant (a medication for insomnia, 5 mg/day before sleep) about half a year. He also had a history of coronary artery bypass grafting (CABG) for myocardial infarction 15 years prior and was taking aspirin (100 mg/day). The patient had no history of allergies. Upon physical examination at the initial visit, the patient’s height was 163.2 cm and he weighed 69.2 kg. His pulse rate was 72 beats/min, blood pressure was 111/81 mmHg, and body temperature was 36.4°C. Ulcers associated with angiosarcoma of the head were observed in the parietal and posterior parts of the left auricle, and facial nerve palsy developed on the left side of the face. The skin rash did not worsen; however, the patient complained of a tingling sensation from the left upper back to the left upper extremity, and allodynia of the left upper arm was evident. Laboratory tests at the onset of postherpetic neuralgia revealed slight anemia with low albumin, but no other findings of note. No significant deviations from the normal range or hepatic or renal dysfunction were observed (Table 1).

**Table 1:** Laboratory data at the first hospitalization. CRP: C-reactive protein; BUN: blood urea nitrogen; AST: aspartate aminotransferase; ALT: alanine aminotransferase; CK: creatine kinase; ALP: alkali phosphatase; γ-GT: γ-glutamyltransferase

Laboratory test	Actual result	Normal range
White blood cell count	5.6×10^3^/μL	3.3-8.6×10^3^/μL
Hemoglobin	12.2 g/dL	13.7-16.8 g/dL
Platelet	278×10^3^/μL	158-348×10^3^/μL
CRP	0.03 mg/dL	0-0.14 mg/dL
Natrium	141 mmol/L	138-145 mmol/L
Potassium	4.6 mmol/L	3.6-4.8 mmol/L
Chlorine	107 mmol/L	101-108 mmol/L
Calcium	8.8 mg/dL	8.8-10.1 mg/dL
BUN	20 mg/dL	8-20 mg/dL
Creatinine	0.84 mg/dL	0.65-1.07 mg/dL
Total protein	6.1 g/dL	6.6-8.1 g/dL
Albumin	3.9 g/dL	4.1-5.1 g/dL
Cholinesterase	174 U/L	240-486 U/L
Total bilirubin	1.0 mg/dL	0.4-1.5 mg/dL
AST	23 U/L	13-30 U/L
ALT	23 U/L	10-42 U/L
CK	34 U/L	59-248 U/L
ALP	46 U/L	38-113 U/L
γ-GT	24 U/L	13-64 U/L
Blood sugar	119 mg/dL	73-109 mg/dL

**Figure 2:**
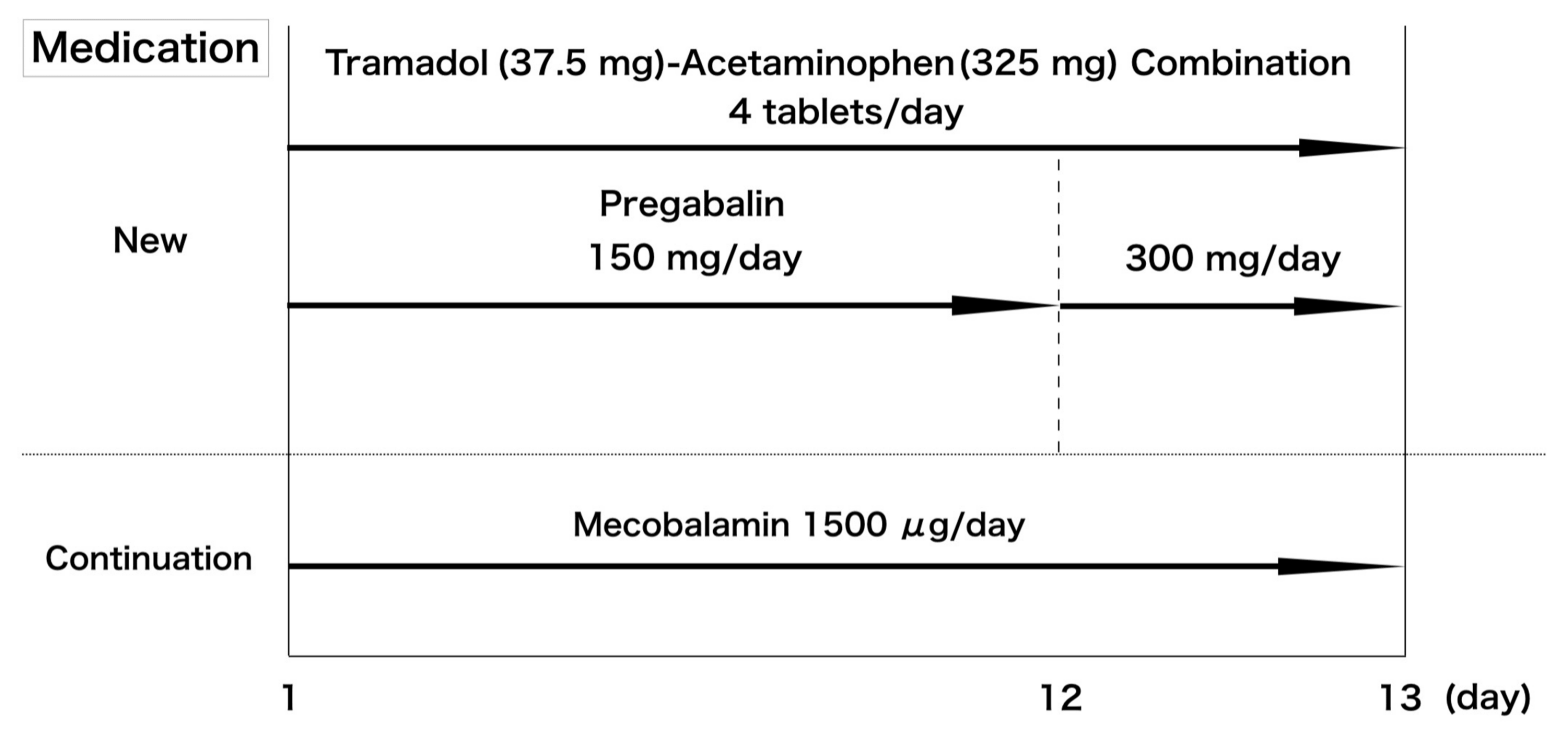
Medication during the first period of hospital admission. Mecobalamin was continued without changing the dose and pregabalin was administered along with a combination of tramadol and acetaminophen. On day 12 of hospitalization, the pregabalin dose was increased to 300 mg/day, and the patient was discharged on day 13.

We admitted the patient to our hospital for pain control, and treatment was commenced with mecobalamin (1500 µg/day), pregabalin (150 mg/day), a combination of tramadol (37.5 mg) and acetaminophen (325 mg) (4 tablets/day) and acetaminophen (600 mg as needed). As there was no renal dysfunction, the pregabalin dose was increased to 300 mg/day, 12 days after admission. The patient was discharged on day 13 after symptom relief without abortive medications (Figure 2).

**Figure 3:**
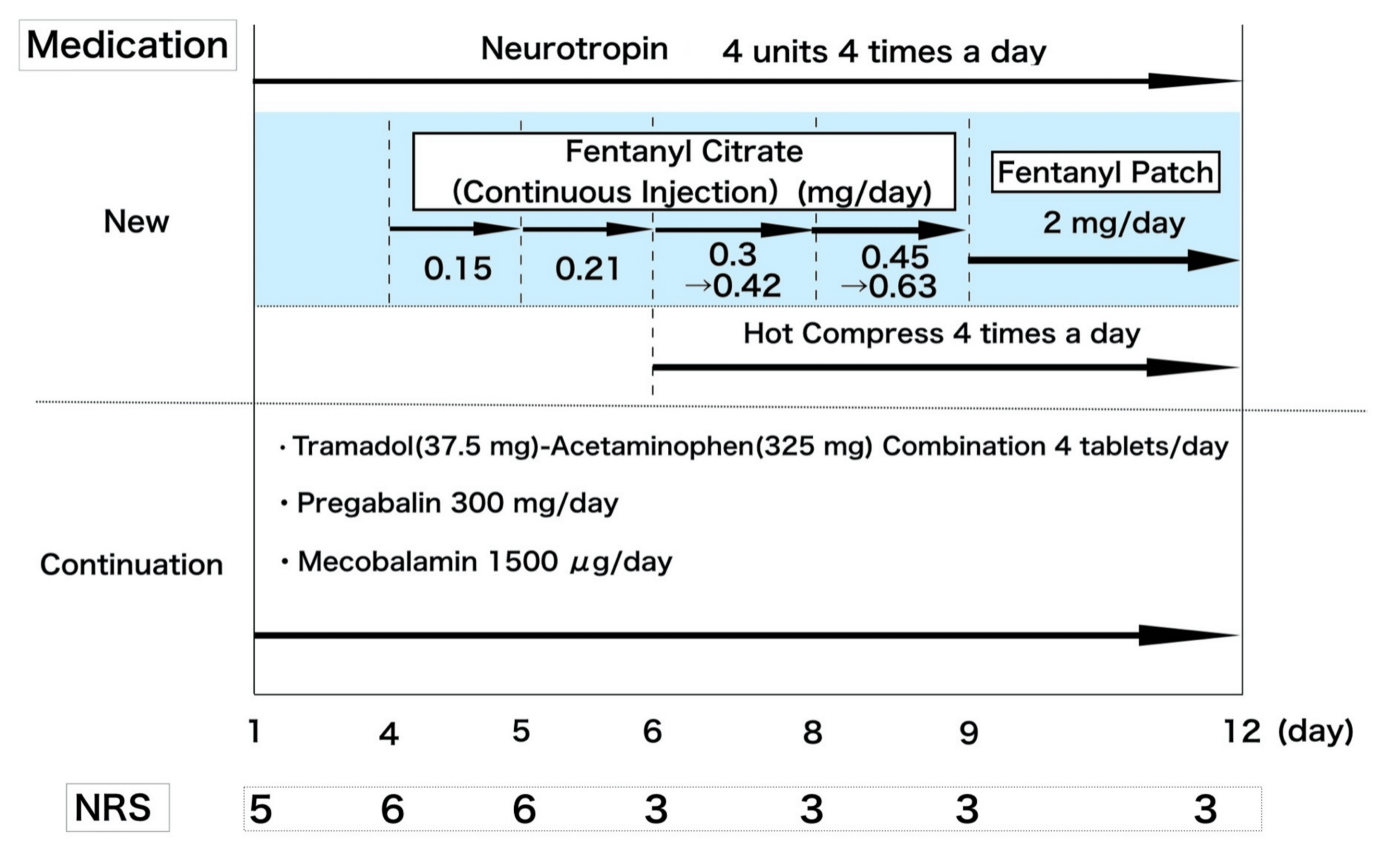
Medication during the second period of hospital admission. The introduction of opioids on day four after admission, along with the use of a hot compress on day six, was noteworthy.

However, the patient’s symptoms recurred. Celecoxib (a non-steroidal anti-inflammatory drug {NSAID}, 200 mg/day) was started but his condition did not improve. Sixteen days after discharge, the patient was readmitted to our hospital for pain management. The location of the pain did not change from the upper back to the left upper arm, which was consistent with the previous hospitalization, and the marked allodynia centered on the left elbow remained unchanged. These skin findings were consistent with those observed during the previous admission. Owing to the pain, the patient had difficulty sleeping at night. Duloxetine hydrochloride was considered; however, we believe that it would be challenging to combine this drug with escitalopram oxalate because duloxetine hydrochloride is a serotonin-noradrenaline reuptake inhibitor (SNRI) with similar efficacy to escitalopram oxalate. Instead, we added four tablets (4 units/tablet) of Neurotropin (an extract from inflammatory rabbit skin inoculated with vaccinia virus) per day and changed the abortive medication to loxoprofen sodium hydrate (60 mg/dose). The patient reported that the intervals between pain exacerbations gradually increased and that he was sleeping better than before his readmission. Although the patient's symptoms resolved, relief was temporary. On day four of hospitalization, the patient reported, “I'm at my limit. I cannot sleep. I had difficulty sleeping and eating,” and his symptoms worsened again. Given the difficulty in controlling pain with oral medications, we decided to initiate continuous opioid infusion. We commenced a continuous injection of fentanyl citrate (0.15 mg/day), gradually increasing the dose while monitoring the symptoms. A hot compress was also used four times a day, covering the painful area directly with a warm towel, starting two days after commencing fentanyl citrate. Figure 3 depicts the continuous medications provided by the outpatient clinic and new medications administered after admission. We increased the fentanyl citrate dose to 0.63 mg/day; following which the patient experienced reduced pain and was able to sleep at night. The degree of pain experienced by the patient was rated on a numerical rating scale (NRS) (Figure 3). Subsequently, fentanyl citrate was switched from continuous infusion to a patch (2 mg/day: 83.3 μg/h), and the patient was discharged on day 12 of hospitalization.

**Figure 4:**
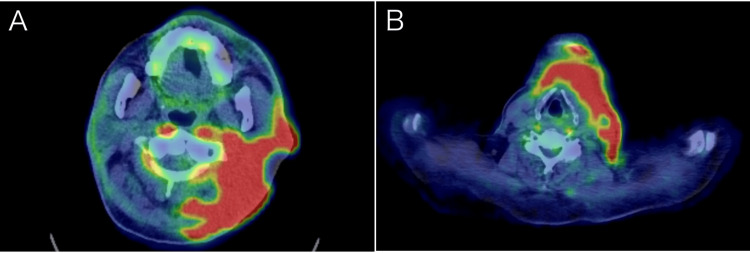
An 18F-fluorodeoxyglucose positron emission tomography (FDG-PET) performed just prior to the third period of admission. (A) Left-sided head and (B) left anterior neck showing strong accumulation of FDG from the skin to deeper tissues.

Twenty-four days later, the patient was hospitalized for a third time because of pain exacerbation. The patient experienced constant pain and difficulties in daily life. An 18F-fluorodeoxyglucose positron emission tomography (FDG-PET) performed five days earlier showed strong uptake of FDG in a wide area in the head (Figure 4A) and neck (Figure 4B), indicating disease progression.

**Figure 5:**
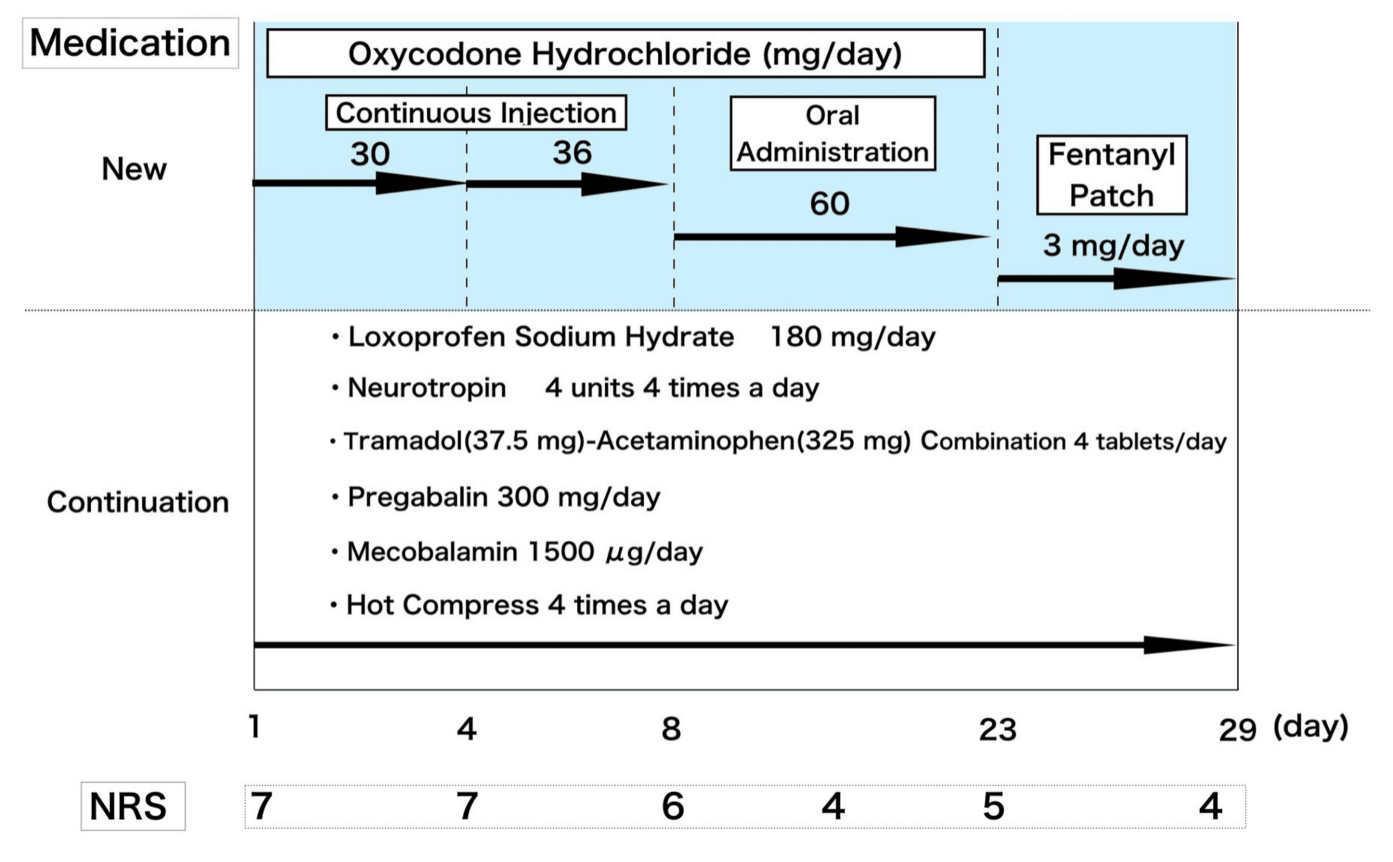
Medication during the third period of hospital admission. Five medications and one non-pharmacological intervention were administered. Continuous injections of oxycodone hydrochloride were administered and switched to oral medications when pain control was achieved. The patient was eventually switched to a fentanyl patch and discharged.

Prior to admission, the patient had commenced taking loxoprofen sodium hydrate (60 mg three times a day) in combination with a tramadol-acetaminophen combination (two tablets four times a day); Neurotropin and pregabalin were continued at the same doses. However, the pain from the cancer worsened. Consequently, the fentanyl patch (2 mg/day: 83.3 μg/h) was switched to a continuous injection of oxycodone hydrochloride (30 mg/day). Changes are made based on general conversion ratios (https://hpal.medindex.co.uk/c/a/opioid-conversion/41). On day four after admission, the oxycodone hydrochloride dose was increased to 36 mg/day (oral morphine equivalent to 72 mg/day), resulting in excellent pain control. The patient reported a few complaints of pain. On day eight, oxycodone hydrochloride was changed from continuous injection to oral administration (60 mg/day), and the patient was followed up without further drug adjustments. At that time, the patient was in the terminal stage of the cancer and developed delirium and irritability. Although the physical distress was relieved, the patient refused medication. There were concerns regarding the discontinuation of oxycodone hydrochloride. Therefore, on day 23, the treatment was switched to a fentanyl patch (3 mg/day). The course of these treatments and the patient’s pain scale scores according to the NRS are shown in Figure 5. Following this change, there was no exacerbation of pain. On day 29 of the third hospitalization, the patient was transferred to a hospice ward as scheduled.

## Discussion

Shingles are caused by reactivation of the varicella-zoster virus, with an individual lifetime risk of 30% [1]. Postherpetic neuralgia (PHN) refers to neuropathic pain arising from peripheral nerves affected by herpes zoster and is defined as pain that persists for at least 90 days [2]. Approximately 20% of patients with herpes zoster report experiencing some degree of pain three months after symptom onset, often described as a burning sensation or electric shock [1]. These symptoms lead to a reduction in the patient’s quality of life (QOL), affecting both physical functionality and psychological well-being. Currently, there is no established treatment for PHN and the primary focus of clinical management is symptom relief [2,3].

In this case, the patient developed postherpetic neuralgia while being treated for head angiosarcoma. We initiated treatment with common analgesics, including acetaminophen, non-steroidal anti-inflammatory drugs (NSAIDs), a combination of tramadol and acetaminophen, and Neurotropin. When these were insufficient, fentanyl citrate and oxycodone hydrochloride were used to further manage the pain. Furthermore, a combination of non-pharmacological interventions, such as direct warming of the affected area (hot compression), effectively alleviated pain. The patient's mental illness complicated our assessment of his complaints and symptoms, and the progression of his primary illness gradually caused persistent cancer pain, making control challenging. However, a combination of pharmacological and non-pharmacological approaches has proven to be successful. Above all, avoiding invasive procedures such as nerve blocks for patients in the terminal stages of cancer is crucial for preventing unnecessary painful symptoms.

The patient was in and out of the hospital for a short period, possibly because he had difficulty expressing his symptoms and might not have improved, as assessed by the NRS. Previous reports have shown that duloxetine hydrochloride, a serotonin-noradrenaline reuptake inhibitor (SNRI), is effective against neuropathic pain, including postherpetic neuralgia [4-6]. However, in this case, the patient had underlying depression and was being treated with escitalopram oxalate, an SSRI, along with other antipsychotics, including aripiprazole, trazodone hydrochloride, and lemborexant, as well as sleep medications. Because of the risk of exacerbating the underlying depression if the medication was altered (such as the development of SSRI withdrawal syndromes, including dizziness, lethargy, and sleep disturbances), we avoided making changes and consulted our psychiatrist regularly [7]. In the absence of an underlying disease, duloxetine should be considered. Pregabalin has also been reported to be effective for treating postherpetic neuralgia and should be considered [8]. Although the dose was limited to 300 mg/day, this effect was observed in our patient. Pregabalin can be increased to 600 mg/day if renal function is preserved, which could have been considered [9,10]. Neurotropin is a non-protein extract isolated from the skin of rabbits inoculated with vaccinia virus and is a commonly used drug for chronic pain, including neuropathic pain, in Japan [11,12]. It was thought to have a certain efficacy in the present case. Acetaminophen and NSAIDs, which are known anti-inflammatory drugs, have proven ineffective in the treatment of PHN [3]. Given the minimal improvement with long-term use of these medications, we recommend considering alternative drugs for similar symptoms in the future. In our case, we used oxycodone hydrochloride and fentanyl citrate as the opioids. The patient reported that oxycodone hydrochloride was effective during his previous hospital admission, and its efficacy against PHN has been documented [13]. However, the effect of fentanyl on PHN has yet to be demonstrated [14]. In this case, the patient experienced difficulty with and refused to take fentanyl, leading us to use a fentanyl patch, although we did not consider it as a first-line drug.

Hot compresses are effective in treating cervical spondylotic radiculopathy and myofascial pain [15,16]. Thermotherapy has also been shown to be effective for herpes zoster and postherpetic neuralgia and should be utilized [17]. We did not perform a nerve block in our patient. No clear evidence that regional anesthesia is effective for postherpetic neuralgia [2], but there is a report that the transversus abdominis plane (TAP) block was effective [18]. Nerve blocks are also invasive procedures and should be considered primarily for patients in good general condition. Eastern Cooperative Oncology Group (ECOG) performance status score for this patient was 4.

## Conclusions

Postherpetic neuralgia is often refractory to treatment, and symptomatic relief remains the primary focus of clinical management. In the present case, the patient's symptoms were effectively managed with a combination of pharmacological treatments and a non-pharmacological approach for symptoms that fluctuated between worsening and remission. Although there is no uniform policy for pharmacotherapy, changes in medications should be considered based on the patient's symptoms. Additionally, non-pharmacological interventions, such as hot compression, are beneficial.
